# Search for Neuro-Endocrine Markers (Chromogranin A, Synaptophysin and VGF) in Breast Cancers. An integrated Approach Using Immunohistochemistry and Gene Expression Profiling

**DOI:** 10.1007/s12022-013-9277-4

**Published:** 2013-11-27

**Authors:** Laura Annaratone, Enzo Medico, Nelson Rangel, Isabella Castellano, Caterina Marchiò, Anna Sapino, Gianni Bussolati

**Affiliations:** 1 Department of Medical Sciences, University of Turin, Via Santena 7, 10126 Turin, Italy; 2 Laboratory of Oncogenomics and Department of Oncological Sciences, Institute for Cancer Research and Treatment, University of Turin, Candiolo, Italy; 3 “Victor Babes” Institute, Bucarest, Romania

**Keywords:** Breast, Neuroendocrine, VGF, Diagnosis

## Abstract

Discordant data are reported in the literature on the definition, incidence and clinical features of neuroendocrine (NE) carcinomas of the breast. This tumour entity is currently assessed by immunohistochemistry (IHC) detecting “general” NE markers such as chromogranin A (CHGA) and synaptophysin (SYP), but other markers have been considered as well. In the present study, in addition to CHGA and SYP, we investigated the expression of VGF, a neurotrophin-inducible gene, which is emerging as a new specific NE marker. In order to evaluate the differential expression of these neuro-endocrine markers in breast cancers, we conducted parallel immunohistochemical and gene expression analyses, using PCR, gene array and real-time quantitative PCR procedures. Data obtained in 28 cases were further validated with a meta-analysis of published datasets of 103 breast cancer cases. The value of IHC positivity (irrespective of the percentage of positive cells) was confirmed by over-expression of the related gene. However, the genetic approach emerged as more sensitive, showing over-expression of NE markers in a subset of IHC-negative carcinomas. In conclusion, the present study confirms, by a novel approach, the occurrence of NE differentiation in breast cancers. Over-expression of one or more NE marker (CHGA and/or SYP and/or VGF) characterizes a significant fraction (approximately 10 %) of infiltrative breast cancers.

**Electronic supplementary material:**

The online version of this article (doi:10.1007/s12022-013-9277-4) contains supplementary material, which is available to authorized users.

## Introduction

The reported incidence of neuroendocrine (NE) differentiation in invasive breast carcinomas (IBC) is variable from 2 up to 20 %, depending on the criteria and detection methods [1–3]. On the basis of morphological and immunohistochemical features, the 2003 World Health Organization (WHO) Classification of Tumors of the Breast defined neuroendocrine carcinoma (NEC) of the breast as a rare subtype of invasive mammary carcinoma showing: (1) presence of morphologic features similar to those of NE tumours of the gastrointestinal tract and lung, and (2) expression of NE markers (chromogranin A and/or synaptophysin) in more than 50 % of tumour cells [3, 4]. The WHO estimated these uncommon tumours as representing approximately 2 % of all breast carcinomas. Cases of IBC with scanty positive cells did not fall into this definition.

In the 2012 revised WHO classification, the NEC definition included three categories: (1) NEC, well-differentiated (carcinoid-like pattern), (2) NEC, poorly differentiated/small cell carcinoma and (3) IBC with NE differentiation determined by immunohistochemistry (IHC) [5]. All of these tumours should express NE markers to a greater or a lesser degree and in this WHO classification the minimum quantity of tumour cells expressing NE markers for defining an IBC as NEC was not specified.

Currently, NE differentiation in breast cancers has been assessed by immunohistochemical procedures detecting “general” NE markers such as chromogranin A (CHGA) and synaptophysin (SYP), and elucidation of specific hormonal peptides has been absent or unproductive [6]. Recently, we have shown the human achaete-scute homolog-1 (hASH-1), a transcription factor that plays a key role in the regulation of mammalian neural and NE cell development, is expressed in NE breast tumours [7].

In the present study, in addition to CHGA and SYP, we investigate the expression of VGF, a neurotrophin-inducible gene, which is emerging as a new specific marker of NE differentiation [8]. The VGF (not acronymic) gene was originally detected on the basis of its regulation by nerve growth factor in rat pheochromocytoma PC12 cells [9] and by brain-derived neutrophic factor and neurotrophin 3 in cultures of neurons [10]. The coded polypeptide of 615 amino acids has a predicted molecular weight of 67 kDa and shares similarities with members of the secretogranin/chromogranin family [11]. This peptide is detectable in subsets of neurons and was found to regulate the hypothalamus–hypophysis–gonad axis [12]. Additionally, VGF peptides have been detected in several types of NE cells within the diffuse neuroendocrine system [13].

In order to evaluate the differential expression of salient neuro-endocrine markers in breast cancers, we conducted parallel immunohistochemical and gene expression analyses in a series of breast cancers. The results indicate that the latter approach is more sensitive and that expression of at least one NE marker in breast cancer is a relatively frequent phenomenon.

## Materials and Methods

### Cases

Twenty-eight invasive breast carcinomas were analysed. A tissue sample (4 mm thick) was fixed in 4 % neutral-buffered formalin (Histo-Line Laboratories, Milan, Italy) at room temperature and embedded in formalin by routine processing (FFPE). In four cases (cases 1–4) one parallel sample (4 mm thick) was embedded in Tissue-Tek® OCT™ Compound, snap frozen in isopentane immediately after dissection and stored at −80 °C. For 24 out of the 28 cases (cases 5–28), Trizol-preserved leftovers from the pre-operative fine-needle aspiration biopsies (FNAB) were also available for gene expression analysis, as recently described [14].

According to the recently updated WHO classification [15], cases were classified as follows: 19 invasive carcinomas not special type (IC-NST), one medullary carcinoma, one infiltrating lobular carcinoma, one tubular carcinoma, two intracystic papillary carcinomas and four cases that fulfilled the 2003 WHO definition of NE carcinomas of the breast by positivity by immunohistochemistry for CHGA and/or SYP in >50 % of cancer cells. Non-neoplastic breast tissue samples obtained from two mastectomies were processed with the same protocol for breast cancer specimens and were used as negative controls.

In addition, we also retrieved two cases of pheochromocytoma of the adrenal gland (fixed and frozen samples), which were used as positive controls.

### Immunohistochemistry

Immunohistochemistry was performed on FFPE tissue sections from each case using an automated slide processing platform (Ventana BenchMark AutoStainer, Ventana Medical Systems, Tucson, AZ, USA) and the following primary antibodies: CHGA rabbit polyclonal antiserum (diluted 1:1,200, Dako GmbH, Hamburg, Germany), prediluted CHGA monoclonal antibody (Clone LK2H10, Roche), VGF rabbit polyclonal antiserum (diluted 1:1,000, Abcam, Cambridge, UK) and prediluted SYP rabbit monoclonal antibody (Clone SP1, Roche). Positive and negative controls (omission of the primary antibody and IgG-matched serum, pheochromocytoma sections) were included for each immunohistochemical run.

### Western Blot Analysis

Western blot (WB) analysis was performed on selected specimens (fresh-frozen samples of two cases of pheochromocytoma of the adrenal gland, one case of NE carcinoma of the breast, two cases of breast IC-NST) and on Jurkat lymphoid cells to check the specificity of the antibody used and of the immunohistochemical reaction.

For VGF expression analysis by WB, fresh-frozen samples and Jurkat lymphoid cells were lysed at 4 °C for 1 h in a lysis buffer (50 mm Tris–HCl, pH 8.3, containing 1 % Triton X-100, 1 mm phenylmethylsulfonyl fluoride, 10 μm/ml leupeptin and 100 units/ml aprotinin). After centrifugation of the lysates at 15,000×*g*, protein contents of the supernatants were measured using the Bradford method. Aliquots containing 30–50 μg of protein per lane were subjected to 10 % SDS-PAGE under reducing conditions and electroblotted onto nitrocellulose membrane filters. The blots were blocked with 5 % non-fat milk in 20 mM Tris–HCl, pH 7.5, 500 mM NaCl plus 0.1 % Tween (TBS-T). The membranes were subsequently incubated overnight at 4 °C with polyclonal rabbit anti-VGF antibody (Abcam, Cambridge, UK) at a concentration of 500 ng/ml. After extensive washing with TBS-T, the blots were incubated for 1 h at room temperature with peroxidase-conjugated protein A (200 ng/ml; Amersham Biosciences), washed with TBS-T, developed with ECL detection reagents (Amersham Biosciences) for 1 min and exposed to X-Omat film (Eastman Kodak Co.).

### RNA Extraction

RNA was extracted from both cytological FNAB samples and frozen histological specimens. RNA extraction from cytological specimens was carried out as previously reported [14]. For frozen histological specimens, we proceeded as follows: the suitability of the material was evaluated by haematoxylin and eosin (H&E) staining; to obtain enough RNA for reverse transcription-PCR, 15 to 20 cryosections were collected from the OCT™ Tissue frozen blocks (20 μm thick) from each specimen. The cryosections were collected into 1 ml of TRI Reagent® solution (Ambion, Inc., Austin, TX) in a 1.5-ml sterile Eppendorf tube and RNA extraction was performed according to the manufacturer’s instructions.

RNA from FFPE tissues was extracted as previously described [16]. RNA pellets were resuspended in DEPC-treated water and quantified by spectrophotometry using a NanoDrop 1000 (Thermo Scientific, Waltham, MA, USA). Finally, RNA samples were stored at −80 °C until further analysis.

### Reverse Transcription

To remove any trace of DNA contamination, 1 μg of RNA from each sample was treated with DNase I (1U/μl; Roche Diagnostics, Mannheim, Germany). To obtain complementary DNA (cDNA), we used the high-capacity reverse transcription kit (Applied Biosystems, Foster City, CA, USA) in a reaction mixture with the following components: 1× reaction buffer, 4 mM dNTPs, 1× random hexamers, 0.5 U/μl RNAse inhibitor, 1.25 U/μl MultiScribe Reverse Transcriptase and DEPC-Treated water. The reaction mixture was incubated at 25 °C for 10 min, at 37 °C for 2 h and finally at 85 °C for 5 min. RNA samples without reverse transcriptase were reverse transcribed and used as negative controls for DNA contamination in PCR analysis. Reverse transcription products (cDNAs) were stored at −20 °C.

### PCR Procedure for CHGA, SYP and VGF

The cDNA samples were amplified to assess chromogranin A (CHGA—N M_001275.3), synaptophysin (SYP—NM_003179.2) and neurosecretory protein VGF precursor (VGF—NM_003378.3) gene expression, using the touchdown-PCR conditions first reported by Korbie and Mattick [17]. CHGA, SYP and VGF PCR were independently performed in a 50-μl reaction volume using the following components: PCR reaction buffer (1× final), MgCl_2_ (1.5 mM final), dNTPs mix (200 μM final), primers (each 0.5 μM final), Taq DNA polymerase (1.25 U final) and distilled water (dH_2_O). Primers, designed using the Oligo explorer 1.5 Software, spanned two different exons resulting in a target sequence of 272 bp for CHGA (primer sequences: Fw. 5′-GCTCCAAGACCTCGCTCTCC-3′ Rev. 5′-CCTGATTGTTCCCCTCAGCCT-3′), 349 bp for SYP (primer sequences: Fw. 5′-GTGCTGCAATGGGTCTTCG-3′ Rev. 5′-CCGTGGCCAGAAAGTCCAG-3′) and 393 bp for VGF (bases 184 to 576; primer sequences: Fw. 5′-TCGTGACACCAGCTGTCTCC-3′ Rev. 5′-GCACGGTCTCGGTCAGCAGA-3′). The reactions were performed on a PTC-100 Peltier Thermal Cycler (MJ Research, Inc., MA, USA) and PCR products were separated by electrophoresis on a 2 % agarose gel-stained with ethidium bromide. To reduce the risk of contamination from previously amplified products, separate lab areas and equipment were used for RNA isolation, amplification and electrophoresis.

### Real-Time Quantitative PCR

We tested the recently described real-time quantitative PCR (RTq-PCR) approach [16] using customized arrays with 24-wells plates devised for investigating expression on NE markers, comprehensive of CHGA and SYP. In three cases on NE carcinomas (cases 1–3), RNA extracted from both, fresh frozen and FFPE material was employed as described [16]. RNA extracted from FFPE material was pre-amplified following the suggested procedure.

### Gene Arrays

Microarray gene expression profiling analysis was performed using RNA extracted from FNAB leftover material available for 24 of the 28 cases [14]. Briefly, biotinylated cRNA was prepared using the Illumina TotalPrep RNA Amplification Kit (Ambion, Inc., Austin, TX) beginning with 500 ng of total RNA and following the manufacturer’s recommendations. Hybridization of the cRNA to the HumanHT-12_V4 Expression BeadChip (Illumina, Inc., San Diego, CA), washing and scanning were performed according to the Illumina BeadStation 5006 manual (revision C). Microarray data were summarized and cubic-spline normalized with the GenomeStudio software (Illumina Inc., San Diego, USA) and subsequently analysed using Excel (Microsoft). The entire microarray dataset is MIAME compliant. Raw and normalized expression data are deposited in Gene Expression Omnibus (GEO Accession number GSE27175). Log(2) transformed expression data were extracted for the genes of interest and then employed, together with detection *p* values, for visualization or for definition of sample positivity.

### Meta-analysis of Published Gene Expression Data

To validate our results in a large cohort of breast cancer patients, we focused on a published gene expression dataset obtained using Affymetrix DNA microarrays on 103 biopsies of aggressive breast carcinomas that were subjected to neoadjuvant treatment [18]. Gene expression data were downloaded from Gene Expression Omnibus (GEO ID: GSE22093) and analysed with Microsoft Excel.

## Results

### Expression of CHGA and SYP

**Fig. 1:**
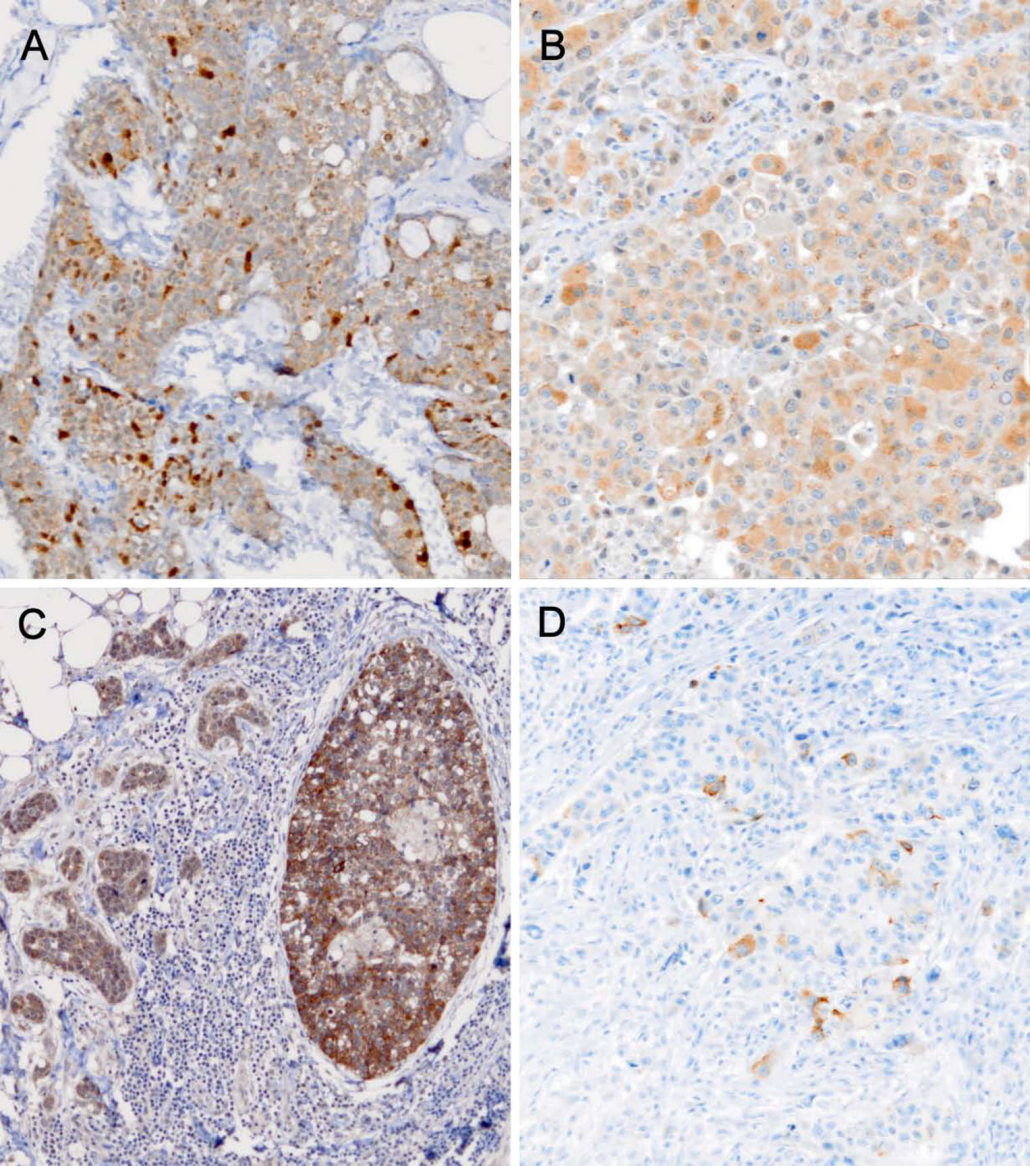
IHC for VGF. **a** A NE breast cancer (case 2) shows IHC expression of VGF of variable intensity in the vast majority of cancer cells. **b** VGF positivity in an invasive carcinoma, no special type (IC-NST) breast cancer (case 5) displaying solid histological patterns. **c** The in situ (*right*) and invasive glandular component in case 10 have different positivity for VGF, which is more intense in the former. **d** Immunohistochemical staining for chromogranin A shows scattered positive cells. The tumour (case 28) showed gene expression positivity for the same NE marker (×100, nuclei counterstained with Haemalum)

The four cases of NE carcinomas of the breast (cases 1–4) showed IHC positivity in >50 % of neoplastic cells for SYP (all cases) and CHGA (cases 1, 2 and 4, but not case 3 which was negative). Moreover, scattered neoplastic cells were found positive for CHGA in cases 5 and 28 (Fig. 1) while for SYP in cases 8 and 26.

**Fig. 2:**
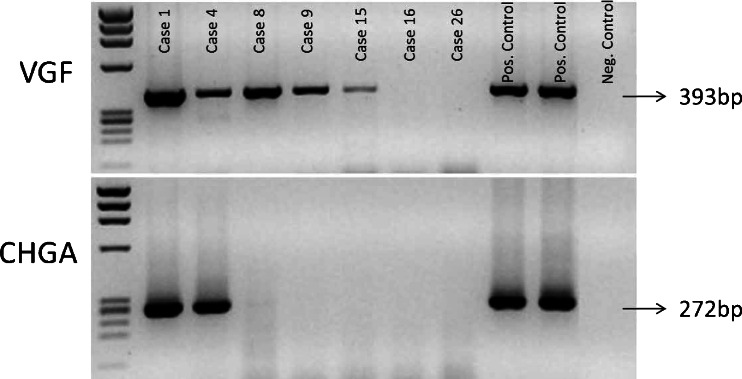
RT-PCR for chromogranin-a (CHGA) and VGF in NE (cases 1 and 4) and non-NE samples (cases 8, 9, 15, 16 and 26). Two cases of pheochromocytoma of the adrenal gland were used as positive controls

**Fig. 3:**
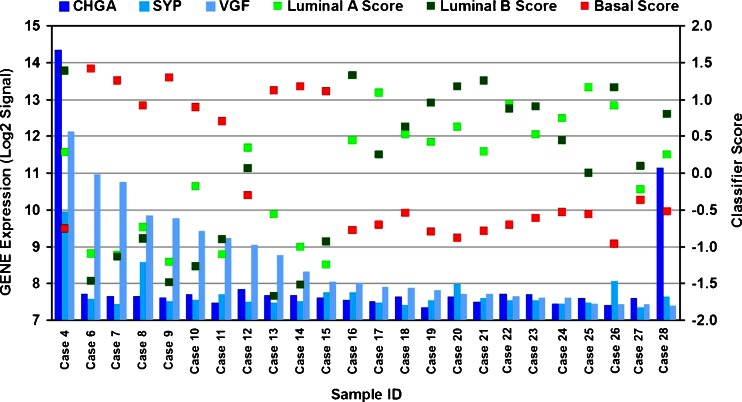
In the waterfall plot, the 24 samples are sorted *from left to right* according to decreasing levels of VGF mRNA (*light blue columns*, *left y-axis* scale). For each sample, the *coloured squares* represent one of the three transcriptional scores (*right y-axis* scale): luminal a (*green*), luminal b (*dark green*) and basal (*red*). *Blue and dark blue columns* represent SYP and CHGA, respectively. Except for the sample expressing the highest VGF levels, carrying a luminal B profile, samples displaying high levels of VGF (above a log2 signal of 9) have a basal-like profile

A high level of gene expression for CHGA and SYP was detected in all IHC-positive cases, irrespective of the number of positive cells. Significant discrepancies in the expression of these two markers were observed in five cases (N. 3, 8, 20, 26, 28; Figs. 2 and 3).

**Fig. 4:**
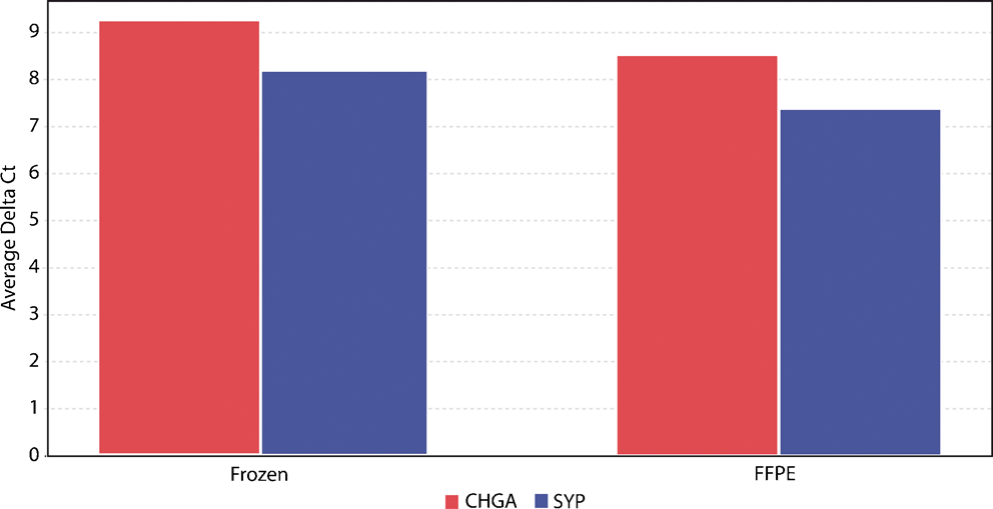
Plot of the average delta Ct matching by `q-PCR, in three cases of NE carcinomas of the breast (cases 1, 2 and 3), the RNA extracted from fresh frozen and FFPE material. The results, confirmed also by scatter plot analysis, show the suitability of FFPE samples for the assessment of CHGA and SYP gene expression

Concordant results were obtained with PCR and gene array procedures. Using the RTq-PCR procedure in three cases on NE carcinoma (Fig. 4), similar and compatible results were obtained by either scatter plot or average delta Ct analysis, using RNA extracted from fresh frozen and from archival material, thus proving the validity of FFPE material for detecting NE gene expression. In case 3, which was negative for CHGA in IHC, gene expression for this NE marker was 35,000-fold down-regulated as compared to cases 1 and 2 (Table 1).

**Table 1:** Histopathological and molecular features of the study cohort

Case no.	Histological type	*G*	Gene expression			IHC		
			CHGA	SYP	VGF	ER	HER2	Ki67
1	NE	2	+++	+++	+++	100	0	16
2	Mucinous NE	1	++	+++	+	100	1	2
3	NE	3	−	+++	++	0	0	35
4	NE	2	+++	++	+++	100	0	23
5	IC + mucinous	3	++	−	++	70	3	35
6	IC-NST	3	−	−	++	0	0	43
7	IC-NST	2	−	−	++	4	0	60
8	IC-NST	3	−	+	++	0	0	70
9	IC-NST	3	−	−	++	0	0	70
10	IC-NST	2	−	−	++	5	1	17
11	IC-NST	3	−	−	++	0	3	43
12	IC-NST	3	−	−	++	95	3	16
13	IC-NST	2	−	−	+	0	0	44
14	Medullary	3	−	−	+	0	0	25
15	IC-NST	3	−	−	+	1	0	60
16	Intracystic papillary	1	−	−	−	100	0	20
17	IC-NST	2	−	−	−	95	1	28
18	IC-NST	2	−	−	−	95	0	21
19	IC-NST	1	−	−	−	90	1	28
20	IC-NST	2	−	+	−	100	1	14
21	Intracystic papillary	1	−	−	−	100	1	18
22	IC-NST	2	−	−	−	100	0	9
23	IC-NST	2	−	−	−	95	1	30
24	Tubular	1	−	−	−	95	1	19
25	ILC	2	−	−	−	88	0	11
26	IC-NST	2	−	+	−	95	1	6
27	IC-NST	3	−	−	−	65	3	34
28	IC-NST	3	+++	−	−	95	3	25

*CHGA* chromogranin A, *ER* estrogen receptor, *G* histological grade, *IC-NST* invasive carcinoma not special type, *IHC* immunohistochemistry, *Lum* luminal, *NE* neuroendocrine, *SYP* synaptophsyn, +++ overexpression

### Expression of VGF

**Fig. 5:**
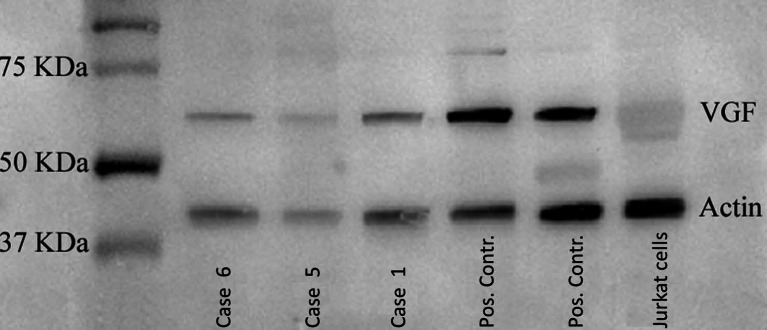
Western blot for VGF. VGF expression analysis by WB performed on two cases of breast IC-NST (cases 5 and 6), one case of NE carcinoma (case 1) of the breast and two cases of pheochromocytoma of the adrenal gland (positive control) and on Jurkat lymphoid cells (negative control)

VGF distribution, as detected by IHC, resulted in a rather weak and diffuse staining in the four cases of NE carcinomas (Fig. 1b). An apparently non-specific staining was detected in some cells in normal ducts, which might suggest a poor specificity of the available antibody. Since the immunohistochemical approach on FFPE tissue section proved poorly reliable, we used WB analysis to confirm the production of the immuno-reactive VGF protein of the predicted MW of 67 kDa in case 1 of NE carcinoma (Fig. 5) as well as in two additional cases which proved positive for VGF gene expression (see below). In the NE carcinoma, a weaker but definite additional band was detected at a higher MW (approximately 80 kDa).

VGF gene expression by RT-PCR and gene array analysis was found in all four NE breast carcinomas that were CHGA and/or SYP positive at the mRNA and protein level (Table 1). The analysis of two non-neoplastic breast specimens gave negative results, whereas two cases of pheochromocytoma expressed by RT-PCR high levels of mRNA for both CHGA and VGF, confirming VGF as a marker of NE differentiation (Fig. 2). Surprisingly, in addition to the four NE carcinomas, VGF was expressed at high level in eight breast carcinomas negative for CHGA and/or SYP (cases 5, 6, 7, 8, 9, 10, 11 and12) and at moderate level in cases 13, 14 and 15 (Figs. 2 and 3). The gene expression profile of these eight cases is strongly positive for VGF (detection *p* < 0.01 and Log(2) signal > 9) when matched with published expression-based classifiers [19] fit with the basal tumour type signature (Fig. 3). Of these, six cases were G3 and two were G2 (Table 1).

The relative expression of CHGA, SYP and VGF is reported in Fig. 3 which shows variation of expression level of the three NE markers in different breast cancers.

### Meta-analysis of NE Markers Expression in Publicly Available Datasets of Breast Carcinomas

**Fig. 6:**
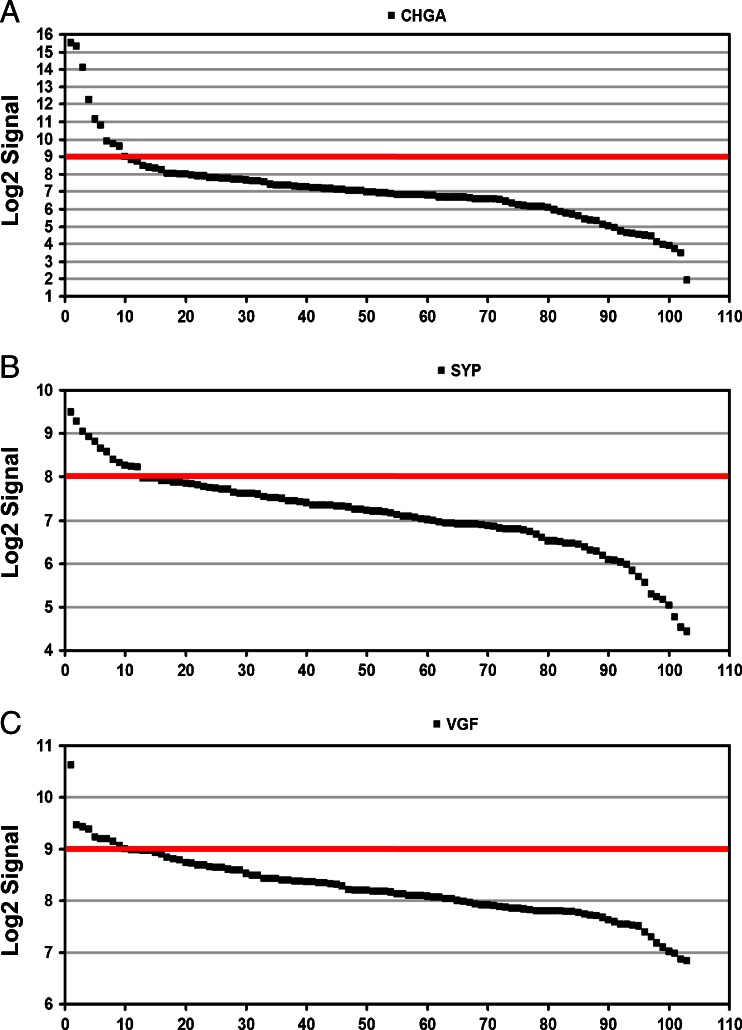
Expression of CHGA, SYP and VGF in 103 breast cancer samples. Waterfall plots showing the expression levels of **a** CHGA, **b** SYP and **c** VGF in 103 breast cancer biopsies. In each panel the samples are sorted by descending levels of the respective gene analysed. The *red lines* indicate the threshold above which samples are considered to express high levels of the gene

**Fig. 7:**
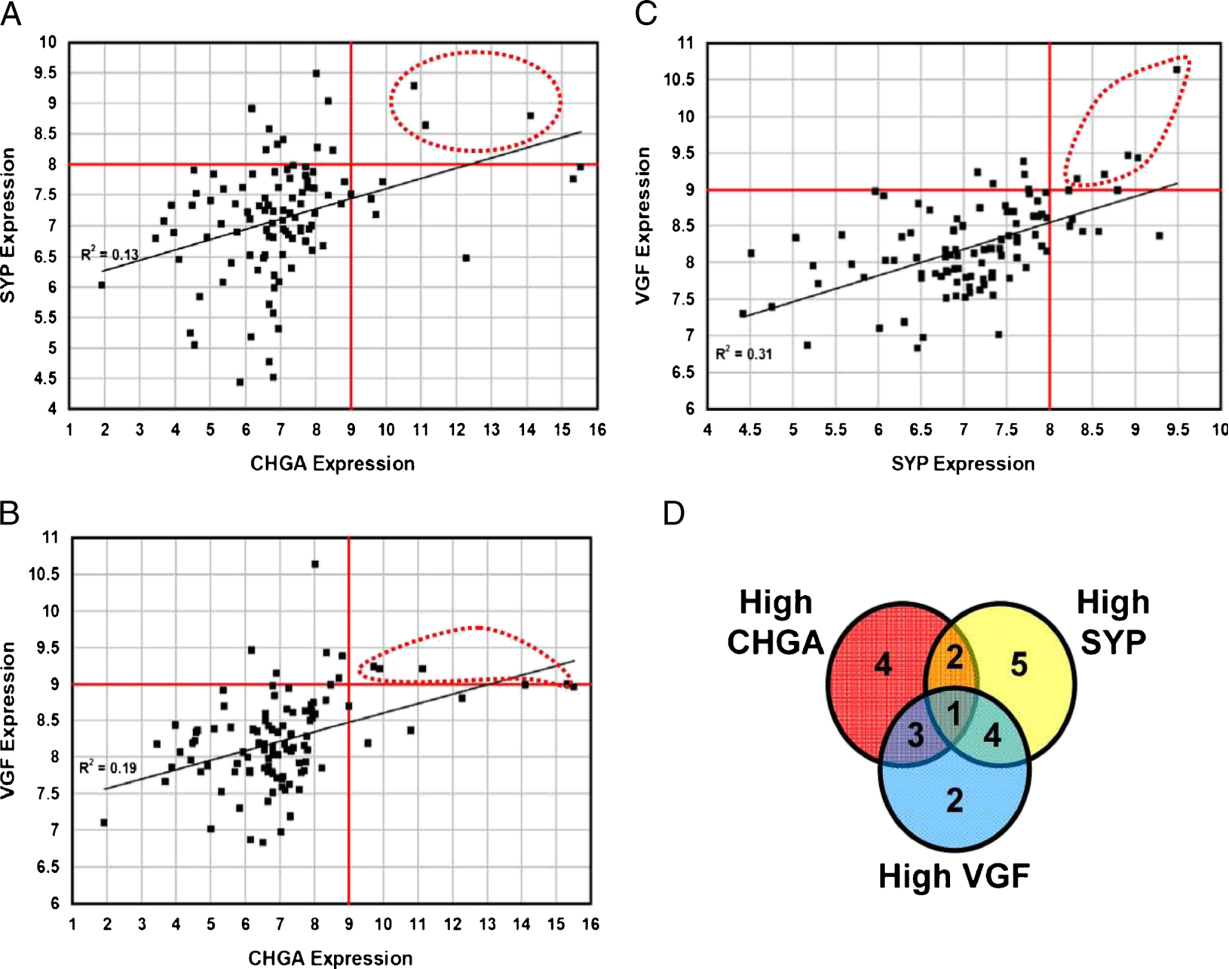
Analysis of correlation between CHGA, SYP and VGF expression in 103 breast cancer samples. Dot plots comparing expression (log2 Signal) of **a** CHGA vs SYP, **b** CHGA vs VGF and **c** SYP vs VGF in 103 breast cancer biopsies. *Red straight lines* indicate the thresholds above which samples are considered to express high gene levels. *Red dotted lines* define samples with high expression of both genes in the panel. **d** Venn Diagram indicating coexpression of high levels of CHGA, SYP and VGF

To check the reproducibility of the observed results, we focused on a published gene expression dataset of 103 breast cancer biopsies originally reported by Iwamoto and colleagues [18]. We evaluated the distribution of CHGA, SYP and VFG expression and found that all three genes have variable expression levels. For each gene, it is possible to define a threshold above which expression can be considered high (Fig. 6). Out of the 103 samples analysed, the Log2 signal is, respectively higher than nine for CHGA in ten samples, higher than eight for SYP in 12 samples and higher than nine in VGF for ten samples. A paired comparison of expression for the three genes displayed an overall correlation, especially between SYP and VGF expression (Fig. 7a–c). Finally, we analysed how many cases had high expression is in two or more genes in the same sample. Interestingly, while there is only one sample with high expression of all three genes, eight of the ten VGF-high samples also had high expression of CHGA or SYP (Fig. 7d). We finally compared expression of the three neuroendocrine marker genes with estrogen receptor and ERBB2 expression, measured at the RNA level by the same microarray experiment. The three genes displayed a concordant behaviour, being expressed at high level preferentially, though not exclusively, in estrogen receptor-positive, ERBB2-negative samples (Supplementary Figures 1 and 2).

## Discussion

The present study is, to our knowledge, the first integrated approach to the definition of NE carcinomas of the breast using IHC and gene expression analysis of specific NE markers. In the four cases (cases 1–4) falling into the proper 2003 WHO definition of NE carcinomas of the breast [3] and showing CHGA and/or SYP protein expression in the majority of cancer cells, gene expression analysis matched the IHC data, thus confirming by an alternative procedure, the existence of a subset of carcinomas of the breast fully oriented towards NE differentiation. In the other 24 cases, a strong expression of at least one NE gene corresponded to a focal positivity or negativity of the related protein. This might be attributed either to a higher sensitivity of the genetic analysis or, less likely, to failure of transduction. The results obtained in our limited series of 28 cases were confirmed by a paired comparison of expression for the CHGA, SYP and VGF genes performed in a publicly available dataset of 103 aggressive breast carcinomas [18] where 10 % of cases showed gene overexpression of at least one NE marker (detection *p* < 0.01 and Log(2) signal > 9) and showed that there is only a partial correlation among these three markers, CHGA expression being often unrelated with that of SYP and VGF.

A significant fraction of breast cancers is characterized by a solid or trabecular arrangement of cells, which may also form pseudo-rosettes and, as remarked by Eusebi and Tavassoli [20], are histologically strongly reminiscent of NE tumours of the gastro-intestinal tract. However, only a minority (approximately 2 %) of cancers can properly be defined as NE carcinomas of the breast as reported in the 2003 WHO “blue” book [3]. In the literature, the incidence of IBC showing evidence of NE differentiation shows ample variation depending on the criteria and detection methods [1–3].

NE differentiation in IBC has so far been achieved through the IHC detection of “general” NE markers, i.e., CHGA and SYP [4, 21–23]. Despite its definite advantages and merits, the IHC approach suffers drawbacks linked to the specificity of the antibodies and to the efficacy of the antigen retrieval procedures. Moreover, the specificity of SYP as an IHC NE marker, despite being improved by the use of high-affinity rabbit monoclonal antibodies, is not absolute, given the reported positivity in adreno-cortical neoplasms [24]. CHGA and SYP are regarded as markers of NE differentiation structurally linked to cytoplasmic granules and vesicles and are, as a rule, simultaneously expressed in typical NETs of different organs such as pheocromocytomas, intestinal and lung carcinoids and pancreatic NETs [8, 25].

In breast cancers, as remarked in the present study, IHC positivity for NE markers is often limited to a single marker and/or to a minority of cancer cells. It might well be argued that this phenomenon might either be related to poor storage of the markers or to a low sensitivity of the procedure. In order to elucidate the issue, we have conducted an integrated approach, using both IHC and gene expression profiling for three “general” NE markers. Although RT-PCR and gene array procedures require the use of RNA extracted from fresh frozen samples, in the present study, we confirmed that the RTq-PCR procedure can reliably be performed on RNA obtained from archival FFPE tissue blocks. This novel approach could complement IHC in the definition of NE differentiation in breast cancers as we have already demonstrated in pancreatic neoplasms [16].

As an additional result, we demonstrated that VGF a recently described NE marker showing similarities with members of the secretogranin/chromogranin family [11] is also expressed in NE breast cancers producing CHGA and/or SYP. In addition, expression of VGF gene was detected in a significant number (10 %) of breast cancers in a large series of aggressive breast carcinomas from a published series [18]. Evidence that VGF gene is over-expressed in a significant number of primary breast cancers was obtained by both PCR and gene array procedures. This is apparently in contrast with the study by Ostrow et al. [26] showing that *VGF* gene was methylated (thus probably silenced) in a vast number of breast cancer cell lines and not in normal tissues.

Finally, discordant data have been reported on the clinical evolution of NEC probably depending on the definition criteria of the case series. According to Wei et al. [27], these tumours represent an aggressive variant, while others maintain that their behaviour does not vary from that of invasive carcinoma NST in relation to grade, stage and expression of estrogen and progesterone receptors (ER and PgR) [2, 4, 6]. In addition, while genetic analyses specifically focused on NE breast tumours allowed to include them among the “Luminal A” or, less frequently, the “Luminal B” subgroup [28], the gene expression profile of most VGF-positive breast cancer classified them as basal. The potential diagnostic and clinical interest of these findings demands further investigations in large cohorts with correlation with clinical outcome.

In conclusion, the gene expression of one or more markers on NE differentiation in invasive breast cancers, as demonstrated in the present study, seems a relatively frequent occurrence whose clinical and biologic significance is presently unknown. The adoption of specifically designed sets of customized arrays could allow the evaluation of expression of genes related to NE differentiation, to tumour aggressiveness and to factors predictive of response to treatment, thus opening the way to selectively tailored treatments.

## Electronic supplementary material

Below is the link to the electronic supplementary material.

ESM 1 (PDF 115 kb)
