# Supplementary material for: Search for Neuro-Endocrine Markers (Chromogranin A, Synaptophysin and VGF) in Breast Cancers. An integrated Approach Using Immunohistochemistry and Gene Expression Profiling
Source: Endocr Pathol. 2013 Nov 27;25(3):219–28. doi: 10.1007/s12022-013-9277-4 (PMC4160574; doi:10.1007/s12022-013-9277-4)
Supplement: Supplementary file 1 — (PDF 115 kb) [file 12022_2013_9277_MOESM1_ESM.pdf]

Supplementary Material

Search for neuro-endocrine markers (Chromogranin A, Synaptophysin and VGF) in breast cancers. An integrated approach using immunohistochemistry and gene expression profiling

Laura Annaratone[1], Enzo Medico[2], Nelson Rangel[1], Isabella Castellano[1], Caterina Marchiò[1], Anna Sapino[1] and Gianni Bussolati[1,3]

[1] Department of Medical Sciences, University of Turin, Torino, Italy

[2] Laboratory of Oncogenomics and Department of Oncological Sciences, Institute for Cancer Research and Treatment, University of Turin, Candiolo, Italy

[3] “Victor Babes” Institute, Bucarest, Romania

Corresponding author:

Prof. Gianni Bussolati

“Victor Babes” Institute, Bucarest, Romania

Department of Medical Sciences

Via Santena 7, 10126 Turin - Italy

Tel.: +390116334274

Fax: +390116635267

gianni.bussolati@unito.it

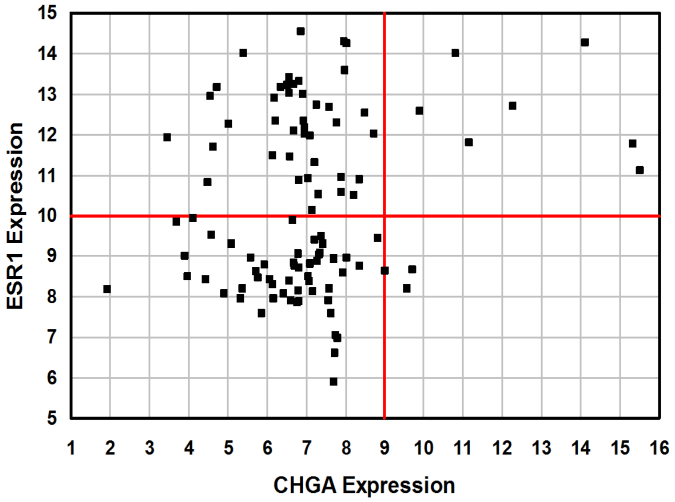

ESR1 Expression
CHGA Expression
15
14
13
12
11
10
9
8
7
6
5
1
2
3
4
5
6
7
8
9
10
11
12
13
14
15
16

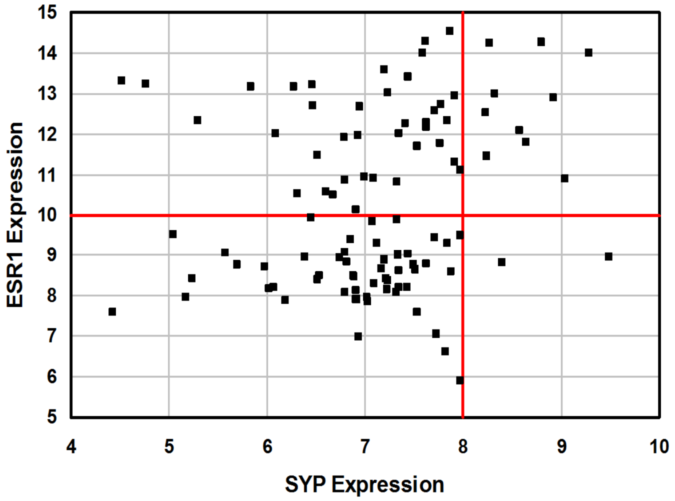

ESR1 Expression
SYP Expression
15
14
13
12
11
10
9
8
7
6
5
4
5
6
7
8
9
10

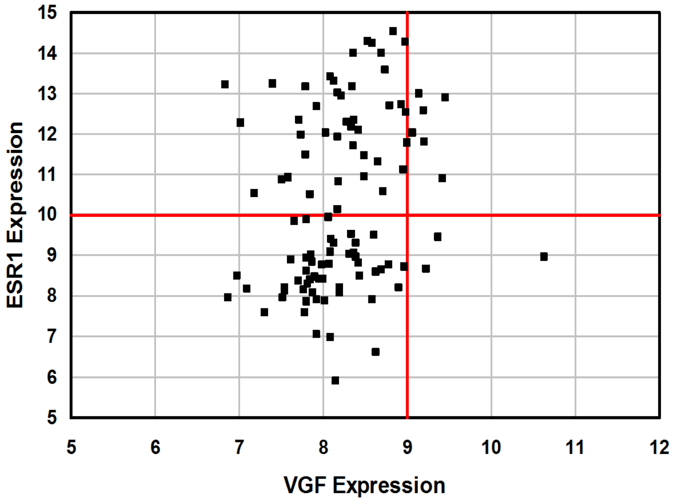

ESR1 Expression
VGF Expression
15
14
13
12
11
10
9
8
7
6
5
5
6
7
8
9
10
11
12

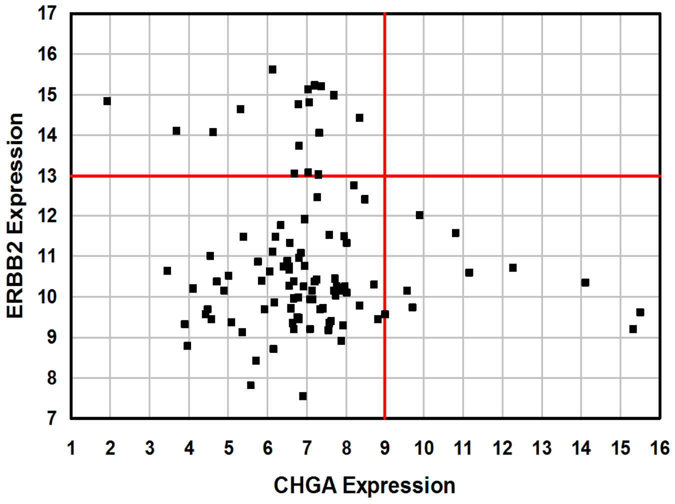

17
16
15
14
13
12
11
10
9
8
7
ERBB2 Expression
1 2 3 4 5 6 7 8 9 10 11 12 13 14 15 16
CHGA Expression

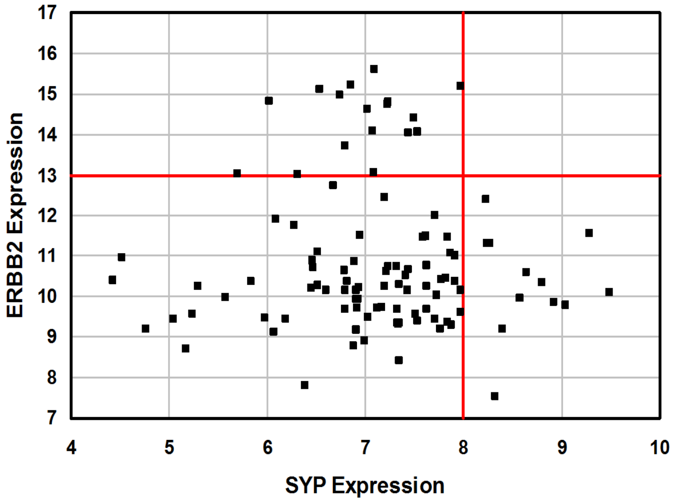

17
16
15
14
13
12
11
10
9
8
7
ERBB2 Expression
4 5 6 7 8 9 10
SYP Expression

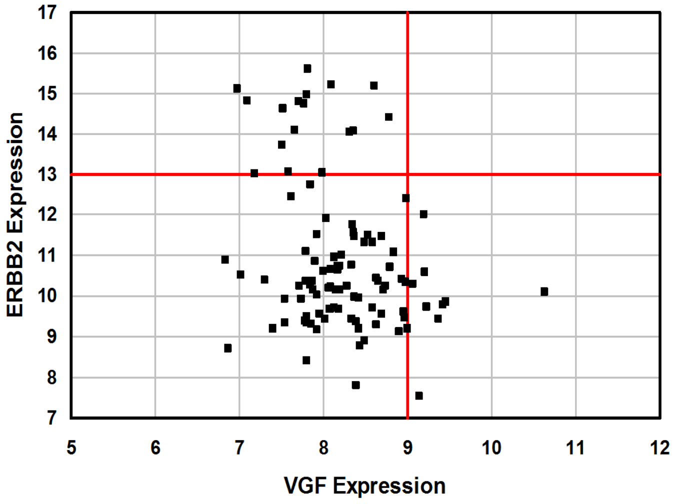

17
16
15
14
13
12
11
10
9
8
7
ERBB2 Expression
5 6 7 8 9 10 11 12
VGF Expression
